# DNA Damage and Pulmonary Hypertension

**DOI:** 10.3390/ijms17060990

**Published:** 2016-06-22

**Authors:** Benoît Ranchoux, Jolyane Meloche, Roxane Paulin, Olivier Boucherat, Steeve Provencher, Sébastien Bonnet

**Affiliations:** Pulmonary Hypertension Research Group, Centre de Recherche de l’Institut Universitaire de Cardiologie et de Pneumologie de Québec, Université Laval, Québec City, QC G1V 4G5, Canada; jolyane.meloche@criucpq.ulaval.ca (J.M.); rpaulin@ualberta.ca (R.P.); olivier.boucherat@criucpq.ulaval.ca (O.B.); Steve.Provencher@criucpq.ulaval.ca (S.P.); Sebastien.Bonnet@criucpq.ulaval.ca (S.B.)

**Keywords:** DNA damage, DNA-damage response, pulmonary hypertension, inflammation, oxidative stress

## Abstract

Pulmonary hypertension (PH) is defined by a mean pulmonary arterial pressure over 25 mmHg at rest and is diagnosed by right heart catheterization. Among the different groups of PH, pulmonary arterial hypertension (PAH) is characterized by a progressive obstruction of distal pulmonary arteries, related to endothelial cell dysfunction and vascular cell proliferation, which leads to an increased pulmonary vascular resistance, right ventricular hypertrophy, and right heart failure. Although the primary trigger of PAH remains unknown, oxidative stress and inflammation have been shown to play a key role in the development and progression of vascular remodeling. These factors are known to increase DNA damage that might favor the emergence of the proliferative and apoptosis-resistant phenotype observed in PAH vascular cells. High levels of DNA damage were reported to occur in PAH lungs and remodeled arteries as well as in animal models of PH. Moreover, recent studies have demonstrated that impaired DNA-response mechanisms may lead to an increased mutagen sensitivity in PAH patients. Finally, PAH was linked with decreased breast cancer 1 protein (BRCA1) and DNA topoisomerase 2-binding protein 1 (TopBP1) expression, both involved in maintaining genome integrity. This review aims to provide an overview of recent evidence of DNA damage and DNA repair deficiency and their implication in PAH pathogenesis.

## 1. Introduction

Pulmonary hypertension (PH) is defined by a mean pulmonary arterial pressure over 25 mmHg at rest and is diagnosed by right heart catheterization. Different groups are defined based on PH etiology. In its most common forms, PH can be due to chronic thromboembolic clots (Group 4), consecutive to left-sided heart or lung diseases (Group 2 and 3 respectively), or due to primary arterial defects (Group 1, called pulmonary arterial hypertension [PAH]) [1,2]. PAH is characterized by a progressive obstruction of distal pulmonary arteries and formation of plexiform lesions leading sooner or later to heart failure. The pathogenesis of PAH is complex and involves pulmonary arterial endothelial cells (PAECs) dysfunction, pulmonary arterial smooth muscle cells (PASMCs) proliferation, apoptosis resistance, metabolic shift (Warburg effect), impaired angiogenesis, phenotypic transition, and chronic inflammation [3,4,5,6,7,8,9,10,11,12,13,14]. Currently, no cure exists for PAH and most therapies targeting vasoconstriction, while offering symptomatic improvement and delaying clinical worsening, do not effectively reverse this devastating disease [2,15]. Indeed despite recent improvements in therapies, the estimated survival rate of patients affected by PAH is 50%–70% at 3 years [16]. Therefore, a better understanding of PAH pathogenesis is mandatory to identify new therapeutic targets capable of interrupting the disease process.

Despite a poor knowledge of the events occurring in early stages of PAH, mounting evidence indicates that oxidative stress and inflammation significantly contribute to vascular remodeling by promoting exaggerated contractility and proliferation of vascular cells [17,18]. These factors are also known to favor DNA damages. Indeed, the DNA sequence can be altered by error-prone DNA polymerases during replication or by environmental factors such as mutagenic chemicals, oxidative stress, radiations, and chronic inflammation. If these damages are not correctly repaired, cells accumulate mutations in their genome, which can lead to death by apoptosis or in some cases to an altered phenotype as observed in cancer [19]. Increased environmental factors and/or dysfunctional DNA-damage response mechanisms may therefore promote the emergence of an apoptosis-resistant and hyper-proliferative phenotype implicated in vascular remodeling [20]. The present review provides an overview of recent insights showing that DNA damage contributes to PAH pathogenesis.

## 2. DNA Damage and Repair

DNA is chemically unstable in physiological conditions, like all biological macromolecules, and is vulnerable to hydrolysis, oxidation, and non-enzymatic methylation [21]. In addition to its intrinsic tendency to decompose, DNA lesions arise from endogenous and exogenous genotoxic agents. Endogenous genotoxic substances are produced by cellular metabolism, which is a source of reactive nitrogen and oxygen species (RNS and ROS), estrogen metabolites, and endogenous reactive chemicals such as aldehydes produced by lipid peroxidation [22] or alkylating molecules like S-adenosylmethionines involved in gene expression regulation through physiological DNA methylation [23,24]. Exogenous genotoxic agents refer to environmental events such as exposure to mutagenic chemicals or physical agents like UV or ionizing radiation (e.g., X-rays) [25,26]. Resulting DNA damages can be single-strand (SSBs) or double strand breaks (DSBs), abasic site (also known as AP site (apurinic/apyrimidinic site)), modified bases, bulky adducts, interstrand/intrastrand crosslinks or insertion of intercalating agents [19,26,27,28,29,30,31,32,33].

DNA integrity is constantly threatened. SSBs, which are the most common type of DNA damage, occur more than 10^4^ times per cell per day, only from endogenous DNA insults and spontaneous DNA decay [30,34]. Taken together, the estimated rate of spontaneous DNA lesions is around 10^5^ per cell per day [25]. The fate of cells against constant DNA damage lies on efficient repair mechanisms called DNA-damage response (DDR). DDR involves multiple pathways for rapid detection, signaling and repair of DNA lesions [35,36,37].

### 2.1. Single-Strand Damage

SSBs are the most common DNA lesions. In this type of lesion only one of the two DNA strands has a defect with a missing or damaged nucleotide and altered 5′ and/or 3′ ends at the lesion site [30]. SSBs may results from attack of DNA bases and deoxyribose by ROS or other electrophilic molecules [38]. Three excision repair pathways exist to repair this type of alteration in DNA integrity, which are base excision repair (BER), nucleotide excision repair (NER) and mismatch repair (MMR).

BER is a pathway involved in resolving non-bulky DNA lesions by excising and replacing abnormal or damaged DNA bases (methylated, oxidized or reduced bases). During BER, the incorrect or damaged base is excised by DNA glycosylases then replaced by DNA polymerases and ligases [39,40,41,42,43]. Poly(ADP-ribose) polymerase 1 (PARP1) can accelerate BER. PARP can bind on AP sites obtained following DNA glycosylases excision [30,44,45]. When fixed, PARP1 synthesizes branched chains of poly(ADP)ribose (pADPr) polymers. pADPr allows the recruitment of X-ray repair cross-complementing protein 1 (XRCC1) scaffolding protein in complex with polynucleotide kinase (PNK), DNA polymerase β and DNA ligase III [46,47,48,49]. pADPr polymers can give hundreds of ADPr monomers, which negatively charge the SSB site. Accumulation of negative charges opens the DNA strands, stabilizes them, and therefore facilitates BER repair. It also releases PARP1 from the AP site, which is then restored by Poly(ADP-ribose) glycohydrolase [30,50,51,52].

Pathways involved in DNA lesions detection for the NER mechanism are mainly important for DNA damage induced by UV. They rely on damage sensor Xeroderma pigmentosum complementation group C and other proteins recruited at the lesion site, such as Cockayne syndrome protein [53,54,55]. Mutations in these NER proteins lead to severe diseases like xeroderma pigmentosum, Cockayne syndrome or trichothiodystrophy [56].

The MMR pathway recognizes base-base mismatches and insertion/deletion loops due to partnerless nucleotides that appear during DNA replication [54,57,58,59,60,61,62]. Mutations on genes that code for proteins involved in MMR is linked to hereditary nonpolyposis colorectal cancer hereditary cancers [59,63,64].

### 2.2. Double-Strand Breaks

DSBs leave no complementary strand that can be used as template during repair. They represent a more serious threat for DNA integrity as they can lead to chromosome breaks and translocation. Three major pathways are implicated in DSB repair: non-homologous end joining (NHEJ), homologous recombination (HR), and to a lesser extent microhomology-mediated end joining (MMEJ).

In the classical NHEJ pathway, Ku70/86 heterodimer binds to the broken DNA strands and forms a complex with DNA-dependent protein kinase. After recruiting other proteins to the damaged site, a DNA ligase IV will seal both ends of DNA strands [65,66,67,68,69,70]. An alternative NHEJ pathway also occurs in cells with deficient classical NHEJ. The alternative NHEJ may also implicate PARP1, which is implicated in SSB repair as described above. PARP1 binds at the DSBs site and may recruit the Mre11-Rad50-Nbs1 complex and scaffolding protein XRCC1/DNA ligase III complex to ligate DNA ends. Nevertheless, the alternative NHEJ pathway leads to large deletion of DNA sequences, rearrangements, and chromosomal translocation as well as being involved in cancer cell pro-survival phenotype [66,67,68,71,72,73,74,75,76,77,78,79].

HR is involved in DSBs and interstrand crosslinks repair. It occurs between late S phase and G2 phase of the cell cycle and is a less error-prone repair pathway than NHEJ. The HR begins with a resection step to produce a 3′ single-stranded DNA end. The protein Rad51 interacts with Rad52, BRCA1, and BRCA2 (breast cancer 1 and 2) to create nucleoprotein filaments that drive strand invasion to the homologous one from the partner chromatid in a displacement loop structure. The lesion site is then repaired using the homologous DNA template [68,80,81,82,83,84,85,86,87,88,89]. The choice between NHEJ and HR depends on the cell cycle phase as well as regulatory factors such as p53-binding protein 1 (53BP1) or BRCA1. Thereby it appears that 53BP1 will favor NHEJ whereas BRCA1 will promote HR [88,90,91,92]. Nevertheless, their implication is not well understood as BRCA1 may also play an accessory role in NHEJ [93]. Both 53BP1 and BRCA1 deficiencies have been linked to cancer development suggesting that both HR and NHEJ are required for genome stability [87,94,95,96].

MMEJ relies on microhomologies of 2–20 bp in both DNA strands. This mechanism is still unclear but among others, PARP1 may also play a role in this type of repair [77,97,98,99]. It appears that DNA polymerase θ also promotes MMEJ and inhibits homologous recombination [77,100]. MMEJ is an error-prone DNA repair pathway that favors oncogenic translocations and cancer development [77,98], and overexpression of DNA polymerase θ gene *POLQ* is associated with poor survival [101,102].

## 3. DNA Damage in Pulmonary Arterial Hypertension

### 3.1. Evidences DNA Damage in PAH

First evidences of somatic genetic mutations involved in PAH pathogenesis were reported in 1998 as a monoclonal origin of PAECs found in plexiform lesions in idiopathic and appetite suppressant-associated PAH [103,104]. Moreover, microsatellite instabilities were observed in growth and death regulation genes in PAECs from plexiform lesions [105]. Somatic mutations in PAECs are not specific to plexiform lesions as severe genetic abnormalities were also observed in more than half of PAH patients’ PAECs and in explanted tissues [106]. Federici and colleagues [107] observed chromosomal abnormalities in 30.2% of PAH-PAECs *versus* 5.3% in control PAECs. Interestingly, DNA damage was not specific to the lung vasculature as it was also increased in lymphoblastoid cell lines and peripheral blood cells from PAH patients when compared to control subjects. Increased mutagen sensitivity to etoposide and bleomycin was also observed in peripheral blood mononuclear cells from PAH patients and non PAH relatives compared to controls [107]. These observations support the hypothesis of a predisposed sensitivity to DNA damage induced by the PAH environment that may act as a trigger of the pathogenesis.

### 3.2. Inflammation

Inflammation is one of PAH hallmarks and is strongly associated with its pathogenesis. PAH can occur as a complication of various systemic inflammatory conditions such as lupus erythematosus, scleroderma, mixed connective tissue disease, Hashimoto thyroiditis, Castleman disease, POEMS syndrome, human immunodeficiency virus (HIV) infection, and autoimmunity [108]. In some cases, the use of anti-inflammatory therapies can improve patients’ conditions [109,110,111,112].

Regardless of the associated diseases, inflammation is present around remodeled vessels in PAH patients’ lungs. Indeed, there is accumulation of perivascular inflammatory cells such as B and T lymphocytes, mast and dendritic cells, and lymphoid follicles [6,113,114,115,116,117,118,119]. Inflammation in PAH is also associated with increased levels of pro-inflammatory cytokines, such as IL1-β, IL-2, IL-4, IL-6, IL-8, IL-10, IL-12p70, and tumor necrosis factor α (TNF-α) [120,121,122]. Some cytokines seem to be good indicators of PAH progression like the monocyte chemoattractant protein-1 (MCP-1), which is upregulated in early stage of PAH [123] or like IL-6, IL-8, IL-10, and IL-12 that increase with PAH severity and appear to be markers of poor survival rate [121].

Preclinical data also demonstrate that inflammation is strongly implicated in the development of pulmonary vascular remodeling. Indeed, IL-6 administration or overexpression in rodent is sufficient to induce pulmonary vascular remodeling and to exacerbate chronic hypoxia-induced PH [124,125,126]. Conversely, IL-6 knockout mice are less susceptible to develop PH under hypoxia [127]. Inflammation favors pro-proliferation and pro-survival phenotypes but also DNA damage through increased ROS/RNS levels produced by vascular cells under inflammatory condition or massively released by neutrophils and macrophages recruited at inflammation sites. ROS/RNS damage DNA through DNA base oxidation and deamination, or through lipid peroxidation and base alkylation [128]. Among PAH-associated cytokines, TNF-α is linked to increased oxidative DNA damage in hepatocytes and myocytes, and inflammation-associated cancers via activation of the transcription factor NF-κB (nuclear factor-κB), which promotes cell survival [129,130,131,132]. ROS/RNS and DNA damage also promote directly or indirectly DDR, which induces inflammation in a vicious cycle that is known to promote aging and carcinogenesis [24,128,133,134,135,136,137]. For example, DNA damage induces IL-6 production which promotes survival and proliferation though activation of the JAK1-STAT3 signaling pathway in tumor cells [138,139,140].

Inflammation in PAH may also be modulated by alterations in the bone morphogenetic protein receptor type II (BMPR2) signaling pathway. *BMPR2* loss-of-function mutations increase susceptibility to PAH [141], and BMPR2 pathway alterations are key features observed in PAH, contributing to aberrant inflammatory response through altered cytokines feedback regulation like the one described *in vivo* and *in vitro* for IL-6 in PASMCs [142,143]. For instances, reduced *BMPR2* gene dosage (*BMPR2^+/−^*) in mice elicits a stronger inflammatory response after LPS (Lypopolysaccharide) exposure [144]. Similar results were observed in PAH-PASMCs harboring a *BMPR2* mutation. The LPS inflammatory response in PASMCs isolated from *BMPR2^+/−^* mice and from PAH patients carrying *BMPR2* mutations was associated with a reduced expression of extracellular superoxide dismutase 3 and increased activation of STAT3 [144]. Superoxide dismutase 3 is an antioxidant that prevents oxidative damage and STAT3 was found to be a major signaling component downstream of diffusible factors dysregulated in PAH (like TNF, IL-6 and PDGF-β) and enhancing proliferation and resistance to apoptosis [144,145,146]. In this study, chronic exposure to LPS leads to PH development in of *BMPR2^+/−^* mice but not in controls, whereas PH and increased inflammation were prevented by tempol treatment, a superoxide dismutase mimetic, confirming the vicious cycle of chronic inflammation and oxidative stress in this PH model [144].

### 3.3. Oxidative Stress

Oxidative stress is characterized by an increased production of oxidants species and/or decreased production of antioxidants. It is associated with increased ROS and RNS as well as decreased nitric oxide (NO) bioavaibility. Oxidative stress seems to play a crucial role in PH [147,148,149,150] as it can favor vessel thickening by increasing transforming growth factor-β1 (TGF-β1), vascular endothelial growth factor (VEGF), fibroblast growth factor-2 (FGF-2) [151], and platelet-derived growth factor (PDGF) production [152], as well as by mediating endothelin-1-induced PASMCs proliferation [153]. ROS also upregulate hypoxia-inducible transcription factors HIF-1α and HIF-2α expression [154,155] also implicated in PAH development [156,157]. In addition, oxidative stress can also promote vasoconstriction via increased production of endothelin-1 [158] and thromboxane A2 [159], decreased production of prostacyclin [160,161], and increased hypoxic cytosolic Ca^2+^ concentration in PASMCs [162,163]. In agreement with the crucial role of oxidative stress in the pathogenesis of PAH [164,165,166,167,168], antioxidant therapy was reported to have beneficial effects in animal models of the disease [169,170,171,172].

The oxidative stress observed in PH is produced by both inflammatory and vascular cells. Indeed, nicotinamide adenine dinucleotide phosphate (NADPH) oxidases, which are important sources of ROS, are found in macrophages and polymorphonuclear as well as in PAECs, PASMCs and fibroblasts [147,173,174,175]. In the lung vasculature, NADPH oxidases 1–5 play a crucial role in increasing ROS generation and promoting vascular dysfunction in PH models. Under hypoxia, NADPH oxidases 2 has been linked to EC dysfunction and vascular ROS production [176] and its upregulation and activation have been linked to neointima formation in animal models [177]. NADPH oxidases 4 upregulation in PAH and under hypoxia has been associated to adventitial fibroblasts resistance to apoptosis and adventitial fibroblasts and PASMC proliferation [173,178]. Interestingly, increased level of TGF-β1, as observed in PAH serum, leads to NADPH oxidases 4 upregulation in PASMCs [179,180]. Vascular ROS can also be produced in ECs by endothelial nitric oxide synthase under L-arginine or cofactor (BH_4_) depletion condition. In these cases, “uncoupled” endothelial nitric oxide produces ROS rather than NO [181]. l-Arginine deficiency can be the result of decreased l-arginine production, increased production/activity of arginase or increased analog competition with asymmetric dimethyl-l-arginine (ADMA). Elevated plasma arginase activity was reported in sickle cell disease-associated PH [182] and increased level of ADMA associated to reduced ADMA catabolism by dimethylarginine dimethylaminohydrolase 2 was linked to PH [183,184,185]. Finally, ROS accumulation can be the result of impaired ROS scavenging system. Indeed, one of the major antioxidants implicated, the superoxide dismutase 2, was found down-expressed in plexiform lesions and within the media and adventitia of remodeled small arteries from PAH patients [186]. Moreover, as described above, BMPR2 deficiency has also been associated with reduced expression of antioxidant superoxide dismutase 3 in *BMPR2^+/−^* mice exposed to LPS [144].

Increased oxidative stress leads to inflammation and cell injuries due to oxidation of proteins, lipids and DNA, which is observed in PAH patients [148,187,188,189,190,191]. The major oxidative DNA lesion is produced by oxidation of guanine into 8-hydroxydeoxy guanosine, which can produce mutations after DNA repair by G:C to T:A transversions [192,193,194]. It was recently published that DNA damages observed in PAH-PAECs and PAH-lymphoblastoid cell lines were associated with increased levels of ROS [107].

### 3.4. Anorexigen Drugs and Selective Serotonin Reuptake Inhibitors

The prescription of Aminorex, fenfluramines derivatives and Benfluorex used as appetite suppressants was followed by PAH epidemics [195,196,197]. All these molecules share structural and pharmaceutical similarities with amphetamine derivatives which are also considered to be risk factors for PAH [108,198,199,200,201]. Fenfluramines, amphetamines and derivatives had been reported to induce systemic DNA damages through oxidative stress [202,203,204,205,206,207,208,209,210,211]. Fenfluramine derivatives are also substrates for the serotonin transporter and potent serotonin uptake inhibitors [201,212]. More recently, the use of selective serotonin-reuptake inhibitors (SSRIs) in late pregnancy was associated with an increase in the prevalence of persistent pulmonary hypertension of the newborn [213,214] as well as clinical worsening and increased mortality in PAH patients [215]. Dhalla and colleagues [216] reported a positive association between SSRIs use and PAH. The serotonin and serotonin transporter (5-HTT) are implicated in PAH pathogenesis by promoting PASMC proliferation and vasoconstriction. 5-HTT expression and activity are found increased in platelets and PH lungs. The use of 5-HTT inhibitor reduces the proliferation of PASMCs induced by serum and serotonin [217] and its knock out in 5-HTT^−/−^ mice was reported to attenuate hypoxic PH [218]. Thus, it has been speculated that SSRIs might increase extracellular serotonin levels that affect PASMCs [212]. Indeed PAH patients are more susceptible to serotonin-induced PASMCs proliferation as 5-HTT expression and activity are found increased in platelets and PAH lungs. This predisposition can be explained by long allelic variants of the *5-HTT* gene promoter that lead to increased 5-HTT expression in PASMCs. A study from Eddahibi and colleague [217] reported that 65% of PAH patients presented homozygous long allelic variants compared to 27% of controls. Interestingly, SSRIs are also known to have genotoxic effects in patients and animal models [219,220,221,222,223,224]. Although dysregulation of serotonin synthesis in PAH development is well established, SSRIs implication in early PAH pathogenesis is still debated. In a recent study, Fox *et al.* reported that both SSRIs and non-SSRIs antidepressant treatments are associated with the same increased risk of PAH [225]. Moreover the absence of correlations between the potency of 5-HTT inhibition or the duration of treatment and the risk of PAH development suggest a non-causal association. Thus the authors suggested that depressive symptoms may be a risk factor of PAH as altered serotonin signaling predisposes to both conditions [225]. Interestingly, in addition to deleterious effects of dysregulated serotonin signaling on lung vasculature, it appears that depressive disorder also leads to increased DNA damage and DDR deficiency [226,227].

### 3.5. Alkylating Chemotherapies

Alkylating agents are antineoplastic molecules used to treat several cancers. They react with guanine base of DNA to create covalent bonds [228]. Depending on their structure, these agents can modify one nucleotide (monofunctional alkylating agent) or two nucleotides (bifunctional alkylating agents) which, in this case, can create interstrand DNA crosslinks [229,230]. If not repaired, these DNA alterations lead to cell death. In healthy cells, BER, NER, and MMR pathways can efficiently remove these alterations. However, cancer cells will be heavily damaged because of their high proliferative phenotype and DDR deficiency (less error-correcting capacity). Nevertheless, the nonspecific action of alkylating agents can also induce mutations in healthy cells with rapid division. Alkylating agents are also known to cause severe injuries to hepatic and pulmonary ECs [231,232]. It was recently published that the use of bifunctional alkylating agents used in chemotherapies were associated with the development of pulmonary veno-occlusive disease (PVOD), an uncommon form of PAH both in human and animals [233,234,235]. The use of mitomycin C, was associated with high risk of anal cancer-associated PVOD (3.9/1000 per year) in comparison with the rare incidence of PVOD in the general population (<1/million per year) [234]. This side effect could be explained by selective toxicity of mitomycin C towards cells expressing high level of the mitomycin C-activating enzyme, NAD(P)H:quinone oxidoreductase. Indeed, NAD(P)H:quinone oxidoreductase is overexpressed in various cancers, but also highly expressed in normal pulmonary vascular endothelium [236]. The pulmonary vascular toxicity of cyclophosphamide could be explained by the lack of detoxifying enzymes, such as aldehyde oxidase and aldehyde dehydrogenase [237] and by endothelial sensitivity to cyclophosphamide-induced damage [238,239]. In addition to DNA alterations, it was noted that *in vitro* cyclophosphamide treatment depleted glutathione in hepatic sinusoidal endothelial cells favoring oxidative stress [240,241,242]. PVOD is also linked to occupational exposures to organic solvents such as trichloroethylene also know to induce DNA damages [243,244]. Interestingly, monocrotaline, a plant toxin used to induce PH in rats, becomes active after it is metabolized in dehydromonocrotaline, a bifunctional alkylating agent, that induces vascular damage [245,246,247]. Alkylating agents may therefore damage the vascular endothelium and limit its repair capacity by inhibiting the proliferation of remaining PAECs. This may lead to a delayed pulmonary vascular injury, progressive remodeling, and PAH.

## 4. DNA Repair Mechanisms in PAH Pathogenesis

DDR dysregulation has also been recently identified as a trigger involved in PAH pathogenesis. Meloche *et al.* [248] reported that DNA damage in PAH was associated with PARP1 overexpression in PASMCs due to a decrease in miR-223 expression [249]. PARP1 maintains cell survival in a context of DNA damage but can also lead to increased levels of IL-6, inflammation and apoptosis resistance via miR-204/STAT3 mediated activation of bromodomain-containing protein 4 (BRD4), nuclear factor of activated T-cells (NFAT), and HIF-1α [248,250,251]. PARP1 inhibition by ABT-888 has been shown to reverse PH in two animal models of the disease (monocrotaline- and Sugen/hypoxia-induced PH) [248]. Moreover, as previously described, PARP1 is implicated in MMEJ and alternative NHEJ, which are known to induce errors, DNA sequences deletions, rearrangements, and chromosomal translocation [98,99,252,253,254,255]. Similar observations were made with Pim1 and Survivin, two onco-proteins overexpressed during DDR activation, associated to increased DNA repair [256,257]. Their overexpression in PAH PASMC and monocrotaline rat remodeled arteries was linked to increased apoptosis resistance, proliferation, and inflammation which were attenuated by their inhibition [258,259].

It has also been described that loss of BMPR2, can lead to impaired DNA damage repair [260]. In this article, Li and colleagues [260] reported how downregulation of BMPR2 in PAH PAECs decreased BRCA1 expression and increased susceptibility to DNA damages. BRCA1 expression was found decreased in endothelium from PAH remodeled vessels compared to control ones [260]. As previously described, BRCA1 is implicated in HR and NHEJ, but its role remains unclear. Whole-exome sequencing has recently led to the discovery of mutations in another gene, *topoisomerase DNA II binding protein 1* (*TopBP1*), also involved in PAH susceptibility [261]. Alteration of TopBP1 expression was found *in situ* in PAECs from idiopathic PAH patients’ lungs. TopBP1 is important in maintaining genome integrity by preventing DNA damage during replication [262,263,264]. In this article [261], siRNA knockdown of TopBP1 resulted in increased DNA damage sensitivity and apoptosis in healthy pulmonary microvascular ECs, whereas its restoration using plasmids in idiopathic PAH microvascular ECs decreased hydroxyurea-induced DNA damage and improved cell survival. The link between newly discovered PAH susceptibility genes and DDR strengthens the fact that impaired DNA repair is involved in PAH susceptibility. Interestingly, PH can spontaneously occur in animal models of impaired DDR. It has been reported that *Ku70*^−/−^ mice, that display impaired NHEJ and genome instability, spontaneously develop severe pulmonary vessels remodeling and PAH [265]. Chronic inhibition of p53, also involved in NHEJ, with pifithrin-α was sufficient to induced PH in rats [266]. p53 knockout also increases hypoxia-induced PH in mice [267]. Lastly, activation of p53 pathway by Nutlin-3a treatment was reported to reduce PH in an animal model [268].

Finally, as summarized in a review by Potus *et al.* [269], DDR is complex and its activation can modify micro-RNA pathways that are impaired in PAH [270]. Moreover nuclear DDR also affects, via the nucleus to mitochondria signaling, the mitochondrial function and mitophagy [271].

## 5. DNA Damage: Beyond the Nucleus

Mitochondrial dysfunction has been linked to cancer [272,273] as well as vascular and lung diseases including PAH [274,275,276,277,278]. ECs mainly use glycolysis and do not rely on mitochondrial metabolism. It has been suggested that endothelial mitochondria mainly serve as signaling organelles for hypoxic response, inflammation, apoptosis, and vasoconstriction [275,277,279,280,281]. PAH patients display dysmorphic, hyperpolarized mitochondria, mitochondrial fission, mitochondria–Endoplasmic Reticulum Unit disruption, and metabolic switch from mitochondrial oxidative phosphorylation to cytoplasmic glycolysis (Warburg effect) [277,282,283,284,285,286]. Similar observations of abnormal mitochondria were made in Fawn-Hooded rats, a rat strain with disrupted mitochondria-ROS-HIF-Kv pathway that spontaneously develops PAH [282,287,288]. The use of dichloroacetate, a mitochondrial pyruvate dehydrogenase kinase inhibitor, improves Fawn-Hooded rats-PAH as well as PH induced by chronic hypoxia or monocrotaline [282,289,290] confirming the role of mitochondria dysfunction in PH development. Interestingly, it has been reported that altered BMPR2 expression was also linked to PAECs mitochondrial dysfunction [291,292]. Altered mitochondria is also implicated in PASMCs apoptosis resistance [284,285] and in right ventricle dysfunction that occurs in PAH and monocrotaline-induced PH [293,294,295].

Interestingly, mitochondria are more sensitive to DNA damage, compared to nuclear DNA since they lack protective histones and their DDR mechanisms only rely on BER and MMEJ [296,297,298,299]. Moreover, it has been reported that mitochondrial DNA (mtDNA) damage repair in PAECs was somewhat slower compared to pulmonary venous ECs and microvascular ECs [300] suggesting that mtDNA damage might be implicated in PAH. Furthermore, mtDNA damage has a potential role in diseases associated with increased risk for PAH such as systemic lupus erythematosus [301,302,303,304]. In a recent study, Fetterma and colleagues [304] found that increased mtDNA damage in atherosclerosis and diabetes mellitus was associated with increased arterial baseline pulse amplitude suggesting a link between mtDNA damage and excessive microvascular pulsatility. Sirtuin 3, a mitochondrial protein among others involved in mtDNA repair via 8-Oxoguanine glycosylase 1 [305] is downregulated in PAH patients and monocrotaline-induced PH rat whereas Sirtuin 3 knockout mice spontaneously develop PAH [306]. In human glioblastoma cell lines, Sirtuin 3 depletion increased irradiation-induced oxidative damage to mtDNA [305]. DDR in mitochondria is less understood and differs from nuclear DDR since similar proteins may have opposite effects on DNA integrity as observed with PARP1 [307,308]. While a role of mtDNA damage in the development and progression of PAH is speculated, further investigations aiming to demonstrate the presence and effects of mtDNA damage in PAH cells remain to be performed.

## 6. Conclusions

**Figure 1:**
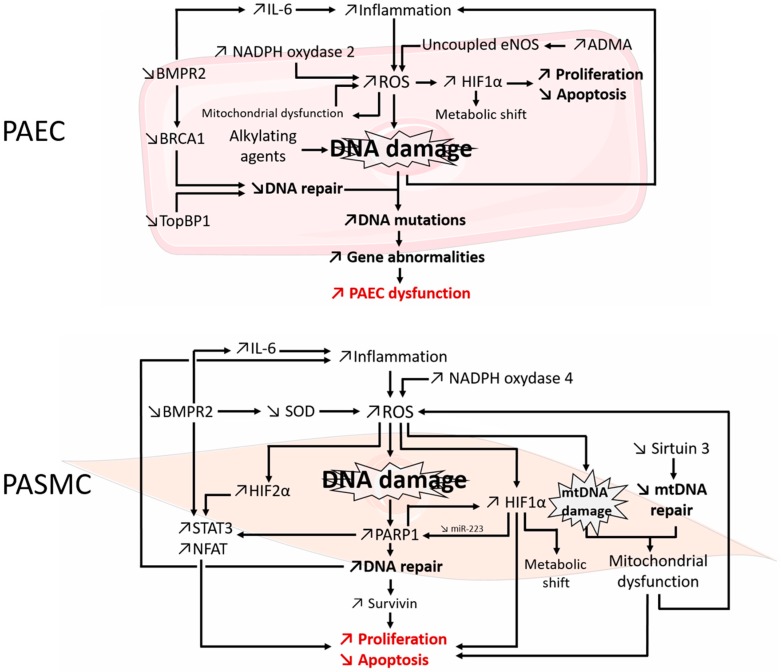
DNA damage and DNA-damage response mechanisms directly or indirectly involved in PAH pathogenesis via PAEC dysfunction and PASMC proliferation and apoptosis resistance (red). PAEC: pulmonary artery endothelial cell; PASMC: pulmonary artery smooth muscle cell; ↗: upregulation; ↘: downregulation.

DNA damage is increased in human PAH lungs, remodeled arteries, PASMCs as well as PAECs. PBMCs also exhibit increased DNA damage, suggesting that this phenomenon is not restricted to the pulmonary vasculature and that intrinsic mutagen sensitivity is present in these patients. Recent studies have found that PAH patients display impaired DNA damage repair associated with TopBP1 and BMPR2-mediated BRCA1 down-expressions. Mutations in *TopBP1* and *BMPR2* genes are associated to PAH predisposition. These DDR alterations lead to genome instability in the PAH environment that favors DNA damage. Indeed the pathogenesis involves chronic inflammation and oxidative stress that are strongly associated with increased DNA damage. In addition, PAH has been linked to drugs such as anorexigen and SSRIs that have genotoxic side effects. Moreover, endothelial DNA damage due to exposure of alkylating agents such as cyclophosphamide or mytomycin C also favors PAH. As in cancer, increased DNA damage and/or impaired DNA repair may promote the proliferative and apoptosis-resistant phenotype that characterizes PAH vascular cells. The implication of DNA damage was also reported in PH animal models reinforcing the observations made in human PAH. DDR mechanisms are complex and interact with cellular pathways that promote directly or indirectly proliferation and apoptosis resistance implicated in PAH development. As described previously for PARP1, DDR also promotes inflammation and therefore DNA damage in a vicious circle. All these evidences summarized in the present review (Figure 1) support the hypothesis that DNA damage sensitivity may act as an early trigger of PAH. Both nuclear and mitochondrial DDR are still not well characterized and crosstalk between them or with other pathological pathways may also be involved in the pathogenesis. Further studies are then required to fully explain how DNA damage and DDR contribute to PAH pathogenesis in order to identify new therapeutic targets.

## Abbreviations

ADMA	asymmetric dimethyl-l-arginine
AP site	apurinic/apyrimidinic site; abasic site
BER	base excision repair
BRCA1	breast cancer 1
DDR	DNA-damage response
DSB	DNA double strand breaks
EC	endothelial cell
HR	homologous recombination
MMEJ	microhomology-mediated end joining
MMR	mismatch repair
NER	nucleotide excision repair
NHEJ	non-homologous end joining
PAEC	pulmonary artery endothelial cell
PAH	pulmonary arterial hypertension
PARP1	poly(ADP-ribose) polymerase 1
PASMC	pulmonary artery smooth muscle cell
PH	pulmonary hypertension
RNS	reactive nitrogen species
ROS	reactive oxygen species
SSB	DNA single-strand break
